# CRIF1 Interacting with CDK2 Regulates Bone Marrow Microenvironment-Induced G0/G1 Arrest of Leukemia Cells

**DOI:** 10.1371/journal.pone.0085328

**Published:** 2014-02-10

**Authors:** Qian Ran, Ping Hao, Yanni Xiao, Lixing Xiang, Xingde Ye, Xiaojun Deng, Jiang Zhao, Zhongjun Li

**Affiliations:** 1 Department of Blood Transfusion, The Second Affiliated Hospital, Third Military Medical University, Chongqing, China; 2 Oncologic Center, The Second Affiliated Hospital, Third Military Medical University, Chongqing, China; University of Turin, Italy

## Abstract

**Background:**

To assess the level of CR6-interacting factor 1 (CRIF1), a cell cycle negative regulator, in patients with leukemia and investigate the role of CRIF1 in regulating leukemia cell cycle.

**Methods:**

We compared the CRIF1 level in bone marrow (BM) samples from healthy and acute myeloid leukemia (AML), iron deficiency anemia (IDA) and AML-complete remission (AML-CR) subjects. We also manipulated CRIF1 level in the Jurkat cells using lentivirus-mediated overexpression or siRNA-mediated depletion. Co-culture with the BM stromal cells (BMSCs) was used to induce leukemia cell cycle arrest and mimic the BM microenvironment.

**Results:**

We found significant decreases of CRIF1 mRNA and protein in the AML group. CRIF1 overexpression increased the proportion of Jurkat cells arrested in G0/G1, while depletion of endogenous CRIF1 decreased cell cycle arrest. Depletion of CRIF1 reversed BMSCs induced cell cycle arrest in leukemia cells. Co-immunoprecipitation showed a specific binding of CDK2 to CRIF1 in Jurkat cells during cell cycle arrest. Co-localization of two proteins in both nucleus and cytoplasm was also observed with immunofluorescent staining.

**Conclusion:**

CRIF1 may play a regulatory role in the BM microenvironment-induced leukemia cell cycle arrest possibly through interacting with CDK2 and acting as a cyclin-dependent kinase inhibitor.

## Introduction

The leukemia resistance to the chemotherapeutic drugs becomes the minimal residual disease (MRD) and is the major obstacle in the treatment of leukemia [1], [2]. The bone marrow (BM) microenvironment is the sanctuary for minimal residual leukemia cells [3]–[5]. In the BM microenvironment, a proportion of the leukemia cells are insensitive to chemotherapy, which is the main cause of the drug resistance and relapse. The BM microenvironment consists of a variety of composition whose roles involved in the leukemia drug resistance remain unclear. It might protect leukemic cells from the killing of the chemotherapeutic drugs by secretion of soluble cytokines, cell adhesion, up-regulating resistance gene and changing cell cycle [6]–[10].

Yoshiyuki et al [11] found that the cell cycle of leukemia cells was arrested at the G0–G1 phase when leukemia cells were co-cultured with BM stromal cells of which the main feature is quiescent [12]. Recent studies have revealed cytokines such as interferon-alpha (IFNα), G-CSF, and arsenic trioxide (As2O3) to be efficient agents for promoting cycling of dormant leukemic stem cells. These activated stem cells become exquisitely sensitive to different chemotherapeutic agents [13], [14]. For instance, histone deacetylase inhibitors in combination with imatinib mesylate can effectively target quiescent chronic myelogenous leukemia stem cells [15]. A growing number of studies have shown that break dormancy of leukemia cells can effectively enhance their sensitivity to chemotherapeutic drugs [16]. Therefore, exploring the mechanism that induces their dormancy would be very important for MRD treatment.

CR6-interacting factor 1 (CRIF1) is a newly discovered cell cycle negative regulatory factor. Previous study in our lab found that the cell cycle of leukemia cells was arrested at G0/G1 phase in vitro bone marrow microenvironment and the expression level of CRIF1 was also increased. Additionally, we found CRIF1 level in leukemia cells co-cultured with bone marrow stromal cells (BMSCs) also increased significantly, suggesting that co-culture induced cell cycle arrest is associated with increased expression of CRIF1 in leukemic cells [17]. We also found that CRIF1 involved in the G1 arrest and thereby the reducing drug-induced apoptosis of leukemic cells which were induced by abnormal BM microenvironment [17] in vitro. Its overexpression increased the percentage of cells in G1 and suppressed growth in NIH3T3 cells [18]. Ito K et al. [19] reported that targeting the quiescent leukemia cells would be an effective way for the future treatment of MRD.

Current treatment of MRD mainly includes chemotherapy, molecular targeted therapy, gene therapy, BM transplantation, and immunotherapy. However, none of them could completely eradicate residual leukemic cells [20], [21]. Therefore, understanding the mechanism of CRIF1 in regulating of cell cycle and activating the quiescent leukemia cells might provide a new treatment strategy to enhance the drug sensitivity of leukemia cells.

## Design and Methods

### 1. Sample sources

Jurkat cell line was purchased from American Type Culture Collection (ATCC). The BM samples used in the study were obtained from the Department of Hematology, Xinqiao Affiliated Hospital of the Third Military Medical University of the Chinese People's Liberation Army (PLA). BM samples were obtained from 40 subjects (15 males and 25 females) with a median age of 46 (range from 6 to 87 years), including 13 healthy, 12 IDA, 7 AML, and 8 AML-CR samples.

This study was approved by the ethical committee on human studies in Xinqiao Hospital, Third Military Medical University, Chongqing, China. All procedures were in accordance with the Helsinki Declaration of 1975 (as revised in 2008). The written informed consent for participation and publication was obtained from each patient or guardian of the child patient.

### 2. Reagents

RPMI1640 medium and fetal bovine serum was purchased from HyClone. The mouse polyclonal to GADD45GIPI (CRIF1) was obtained from Abcam. The goat primary antibody to CRIF1 (sc-103445), rabbit primary antibodies against CDK2, CDK4, CDK6,Cyclin E and D, and Protein G PLUS-Agrose Immunoprecipitation Reagent were purchased from SANTA. RIPA and Trizol were purchased from BeyoECL Plus. The Taq and T4 Ligase were purchased from Takara. ONE-HOUR Complete IP-Western Kits L00232 and L00231 were purchased from GenScript. Plasmids pET-32a/CRIF1 and pLenti6/V5-D-TOPO vector were constructed in our laboratory previously. The CRIF1siRNA was purchased from SANTA (sc-97804).

### 3. Isolation and expansion of BMSCs

The isolation of BM stromal cells (BMSCs) and their co-culture with Jurkat cells were performed as described by Fortney et al [22]. Briefly, 2 ml of BM was mixed 4 ml 1.077 g/L Percoll and centrifuged at 1800 r/min for 15 min. Cells in the middle white layer were harvested and washed with 1× phosphate-buffered saline (PBS, pH 7.4) twice, followed by centrifugation at 1000 r/min for 5 min. Cell pellets were resuspended and cultured in RPMI-1640 medium containing 12.5% FBS, 12.5% horse serum, 1×10^−6^ mol/L hydrocortisone, and 10 ng/mL bFGF in an incubator with 5% CO_2_ at 37°C. Culture medium was half changed at day 3 and full changed at day 5. Cells were passaged at 80% confluence. When the second generation of cells became 80% confluent they were seeded in24-well plates at density of 2×10^4^ cells/cm^2^. After 24-hour adherent culture, 2×10^5^/ml Jurkat cells in exponential phase were added to BMSCs. After 24 hours, co-cultured cells were used for the following experiments.

### 4. Cell culture

Jurkat cells were cultured in RPMI1640 medium supplemented with 10% FBS in an incubator contained humidified 5% CO_2_ and 95% oxygen at 37°C. The Jurkat cells were co-cultured with the second-generation of isolated BMSCs *in vitro*.

### 5. Production of CRIF1 overexpression virus

Plasmid pLenti6/V5-D-TOPO/CRIF1 was constructed by inserting PCR-amplified human CRIF1 cDNA from pET-32a/CRIF1 into the pLenti6/V5-D-TOPO vector. The constructs were verified by restriction enzyme analysis and DNA sequencing. The production, concentration and titration of lentivirus were carried out as described previously [23]. Briefly, 5 ug pMD2.G, 15 ug psPAX2 and 20 ug vectors were co-transfected into 293 cells in a 10-cm plate using calcium phosphate transfection method. The supernatant was replaced with fresh DMEM medium 10 hours later. After additional 48 hours, the viral stocks were collected and filtered through 0.45 um filter. To concentrate the lentivirus, the stocks were centrifuged at 50000 g for 2 hours. The pellet was suspended in an indicated volume of HBSS. To titrate the virus, serial dilutions of virus were added into 293 cells and the titers were calculated by the proportion of viral infected cells, which was measured using FACS.

### 6. Transfection of CRIF1-siRNA into the Jurkat cell line

The 21-nucleotide siRNA targeting the human CRIF1 (GenBank™ accession number AF479749) was purchased from SANTA. The sequences of CRIF1 siRNA are Sense: UGCCACAGAUGAUUGUGAAtt and Antisense: UUCACAAUCAUCUGU-GCAtt. The transfection procedure was performed as described in the manufactural instruction of sc-97804.

### 7. Flow cytometry

The flow cytometry (Beckman Coulter, MoFlo XDP) was used in the study to detect the cell cycle of Jurkat cells. Cells were washed with 1×PBS and then fixed with ice-cold 70% ethanol. Samples were washed with 1×PBS and stained with propidium iodide (60 µg/ml, Sigma) containing RNase 100 µg/ml (Sigma) for 30 min at 37°C, followed by flow cytometry analysis.

### 8. Real-time quantitative PCR

Real-time quantitative PCR (ABI 7500) was used to analyze the mRNA level of CRIF1 in Jurkat cells and 40 BM samples. All reactions were performed in triplicate using 20 µl samples. The reaction protocol was as follows: heating for 5 min at 94°C, followed by 40 cycles of amplification (30 s at 94°C, 30 s at 59°C and 30 s at 72°C). The primer sequences were as follows: CRIF1:5′-GCCGAAGAACGCGAATGGTAC-3′, 5′-AGCGGGCACTCCTTGGGT-3′;β-actin: 5′-ACCCCGTGCTGCTGAC-CGAG-3′, 5′-TCCCGGCCAGCCAGGTCCA -3′.

### 9. Western blot

Western Blot was used to analyze the protein expression of CRIF1 in Jurkat cells and 40 BM samples. Total protein lysates were extracted with RIPA and denatured by boiling. Protein samples were resolved on 12% SDS-polyacrylamide gels and transferred to polyvinylidene fluoride membranes. Membranes were blocked in PBS containing 5% (w/v) nonfat dry milk and 0.1% Tween and incubated for 2 h with appropriate antibodies. Blots were developed using horseradish peroxidase-linked anti-mouse secondary antibody and developed with a chemiluminescent detection system (Phototope-HRP Western blot detection kit; New England Biolabs).

### 10. Co-immunoprecipitation

Jurkat cells were co-cultured with BMSCs for 24 h, and protein extraction of Jurkat cells was performed for co-immunoprecipitation to analyze the interaction between CRIF1 and CDK2, CDK4, CDK6, and Cyclin D and E. Samples containing 500 µg total cellular protein were transferred into a 1.5 ml microcentrifuge tube, and incubated with 1 µg primary antibody at 4°C for 1 h. 20 µl resuspended Protein G PLUS-Agrose was added and incubated at 4°C on a rocker overnight. Immunoprecipitates were collected by centrifugation at 2500 rpm at 4°C for 5 min. The pellet was washed 4 times with PBS, and resuspended in 40 µl of 1× electrophoresis sample buffer, then boiled for 3 min. The analysis of samples were used ONE-HOUR Complete IP-Western Kit L00232, L00231 (GenScript).

### 11. Immunofluorescence

Jurkat cells were washed three times in PBS after co-cultured with BMSCs for 24 h, and fixed with 4% formaldehyde for 30 min at room temperature. Cells were washed three times in PBS and incubated in blocking solution containing 5% bovine serum albumin in PBS. For permeabilization, cells were incubated in 0.2% Triton X-100. CRIF1 and CDK2 antibodies were incubated with the cells overnight at 4°C. Cells were washed five times with PBS and incubated with secondary antibodies conjugated with FTIC or Cy3 for 30 min at 37°C. The cells were washed five times in PBS. For nuclear staining, cells were incubated with DAPI for 2 min, then washed five times in PBS. Fluorescence was visualized using a confocal microscopy.

### 12. Statistics analysis

The expression levels of CRIF1 gene in the tested samples were expressed in the form of cycle threshold (CT) level; then normalized copy number (relative quantitation) was calculated using the ΔΔCT equation as follows: ΔΔCT = ΔCT of BM sample - ΔCT of β-actin, then the normalized copy number (relative quantitation) = 2^−ΔΔCT^. All data were presented in mean ± s.d. and data from multi-group were analyzed using one-way ANOVA followed by post-hoc test. Results were considered significant when P<0.05.

## Results

### Comparison of CRIF1 levels in human BM samples

**Figure 1:**
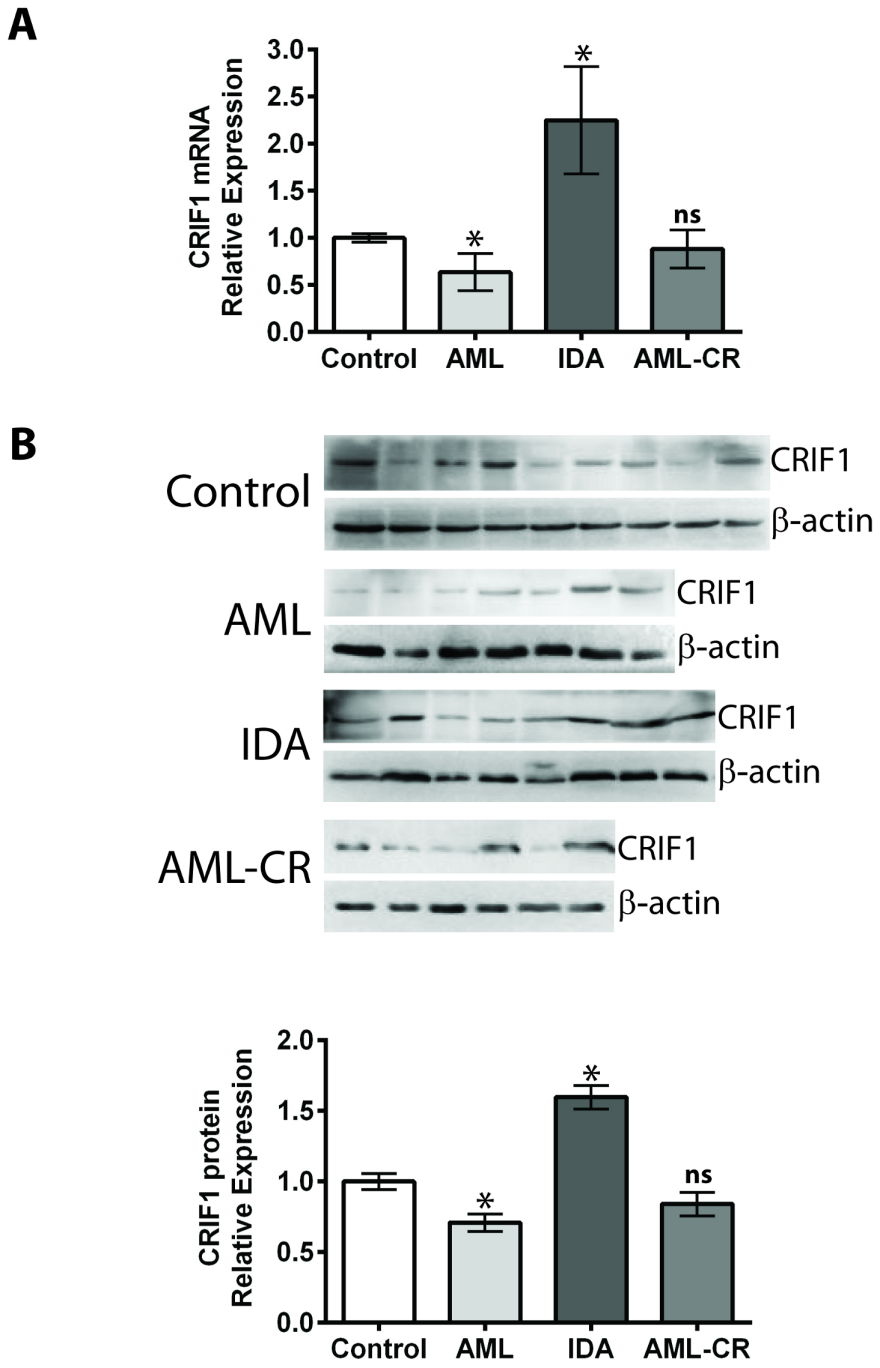
Comparison of the mRNA and protein levels of CRIF1 in human BM samples. (**A**) CRIF1 mRNA levels were examined with real-time PCR. (**B**) CRIF1 protein levels were quantified with WB. *p<0.05, v.s. healthy control group; ns, no significance v.s. control group.

In this study, we compared mRNA and protein levels of CRIF1 in 40 BM samples, including 13 healthy human samples, 7 AML samples, 12 IDA samples, and 8 AML-CR samples. The mRNA level of CRIF1 was detected using real-time quantitative PCR (**Figure 1A**, **Table 1**). Results showed that the AML group had a significantly decreased (0.64 fold, p<0.05) CRIF1 mRNA level compared to the healthy group. IDA patients showed a 2.25-fold higher level of CRIF1 mRNA compared to the healthy group (P<0.05). The mRNA level of CRIF1 in the AML-CR group was not significantly different from that of the control group (0.88 fold, P>0.05). The protein levels of CRIF1 were examined using WB (**Figure 1B**). The changes of CRIF1 protein level among groups showed a similar trend as that of the mRNA expression. Compared to the healthy group, AML group showed a significant decrease (0.70 fold, p<0.05), and the IDA group exhibited a 1.59-fold (p<0.05) increase in CRIF1 protein level. The observation that CRIF1 expression decreased in the AML patients but increased in the IDA patients suggested that expression level of CRIF1 was associated with the different types of blood diseases.

**Table 1:** mRNA levels of CRIF1 in patients and healthy subjects.

	Sample No.	Sex	Age(years)	Relative mRNA ΔCt/AvgΔCt
**Control (n = 13)**	2	F	49	0.578
	17	M	54	1.143
	18	F	38	0.975
	27	F	56	1.173
	20	F	47	1.004
	21	M	69	0.766
	30	M	48	1.104
	31	F	57	1.019
	45	F	47	0.993
	47	F	60	1.127
	39	M	25	1.167
	40	M	58	0.958
	57	F	39	0.992
**IDA(n = 12)**	13	M	75	3.488
	35	F	48	1.536
	46	F	61	1.847
	49	F	40	1.471
	62	M	55	0.415
	3	F	22	1.318
	6	F	43	0.445
	23	M	56	2.480
	34	F	31	1.025
	42	M	50	3.823
	37	F	47	7.545
	38	F	17	1.585
**AML(n = 7)**	22	M	45	0.207
	25	F	87	0.275
	12	F	36	0.160
	11	F	77	0.622
	43	F	19	1.677
	71	F	52	0.805
	72	M	6	0.710
**AML-CR (n = 8)**	61	M	30	1.607
	41	F	19	0.500
	67	F	36	0.472
	58	F	35	0.434
	15	F	30	0.217
	51	M	14	0.793
	36	M	15	1.481
	64	M	24	1.566

### Expression level of CRIF1 affected the cell cycle of Jurkat cells

**Figure 2:**
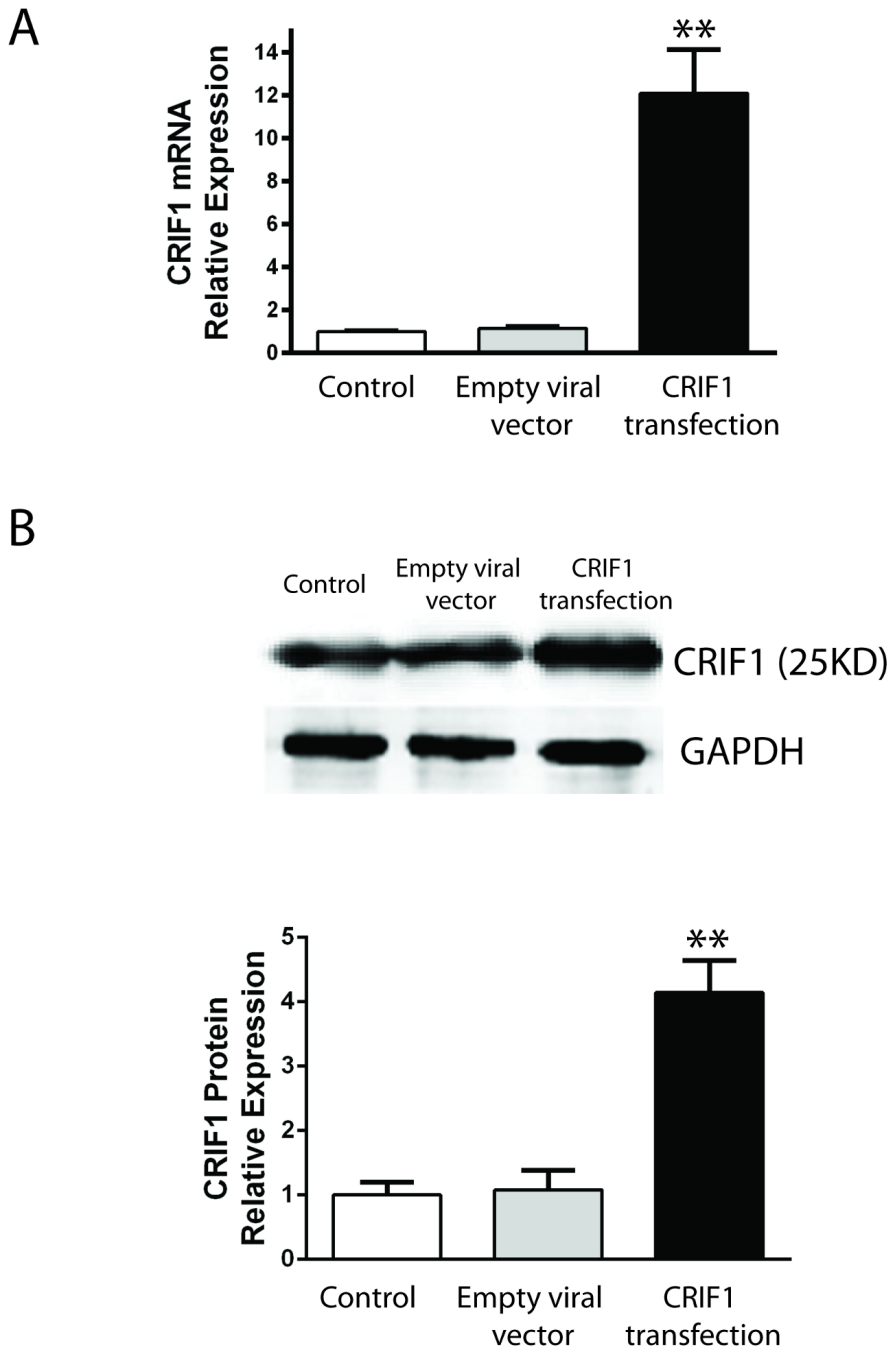
Viral infection mediated over-expression of CRIF1 in Jurkat cells. (**A**) mRNA levels of CRIF1 in Jurkat cells were quantified with real-time PCR. (**B**) Protein levels of CRIF1 were quantified with WB. **p<0.05, v.s. empty viral vector.

**Figure 3:**
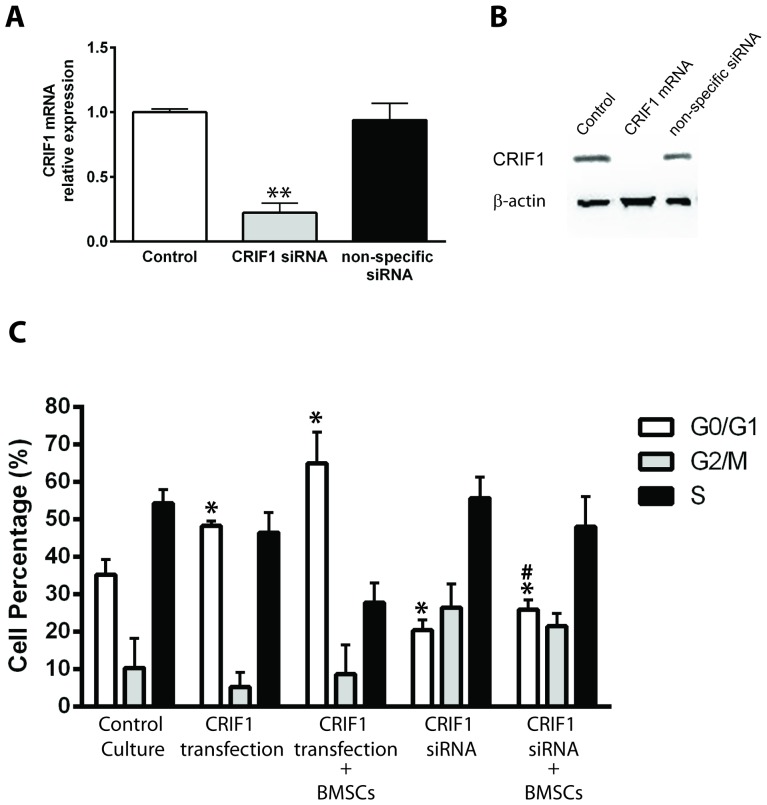
Effect of CRIF1 expression level on the cell cycle of Jurkat cells. The proportion of cells at G0/G1, G2/M and S phase were assessed with flow cytometry and plotted for statistical analysis. *p<0.05, v.s. control G0/G1; #p<0.05, v.s. G0/G1 in cells with CRFI1 overexpression and BMSCs co-culture.

To study whether the expression level of CRIF1 affects the cell cycle of leukemia cells, we used viral vector pLenti6/V5-D-TOPO/CRIF1 to overexpress human CRIF1 in Jurkat cells. The real-time quantitative PCR result showed a 12-fold increase in the expression level of CRIF1 mRNA at 48 hours after viral infection (**Figure 2A**). Correspondingly, CRIF1 protein level showed a 4-fold increase as detected by western blot (**Figure 2B**). To decrease the CRIF1 level, we used siRNA against CRIF1 and the specific down-regulatory effect on CRIF1 mRNA was confirmed with non-specific mRNA (**Figure 3A and B**).

The cell cycles of the control cells or cells with CRIF1 over-expression were analyzed by flow cytometry. The distributions of cells in G0/G1, G2/M and S phases were plotted for quantification (**Figure 3C**). Our result showed that overexpression of CRIF1 in Jurkat cells significantly increased the proportion of cells in the G1 phase (48.3±1.31% vs 35.27±4.04%, p<0.05, **Figure 3C**), suggesting a role of CRIF1 in arresting cell cycle. Co-culture of CRIF1 over-expressed Jurkat cells with BMSCs for 24 h resulted in a further increase of G0/G1 cell population (64.97±8.34%, p<0.05).

To further confirm the role of endogenous CRIF1 in the induction of cell cycle arrest in leukemia cells, siRNA against CRIF1 was transfected into Jurkat cells. In contrast to CRIF1 overexpression, reduction of CRIF1 level in Jurkat cells led to a decrease in the G0/G1 population (20.41±2.81%, p<0.05) but an increase in G2/M (26.48±6.37%, p<0.05). CRIF1 siRNA administration in Jurkat cells also reversed BMSCs co-culture induced G0/G1 cell arrest, showing as a decrease in G0/G1 cell population (25.89±2.71%, p<0.05) and an increase in G2/M cells (21.51±3.45%, p<0.05), indicating that depletion of CRIF1 promoting the cell cycle progress in the leukemia cells. All these results suggested that the expression level of CRIF1 is closely related to the cell cycle of Jurkat and CRIF1 might play a regulatory role in BMSCs induced cell cycle arrest of leukemic cells.

### Identify the protein interacting with CRIF1 in the G1/S checkpoint in Jurkat cells

**Figure 4:**
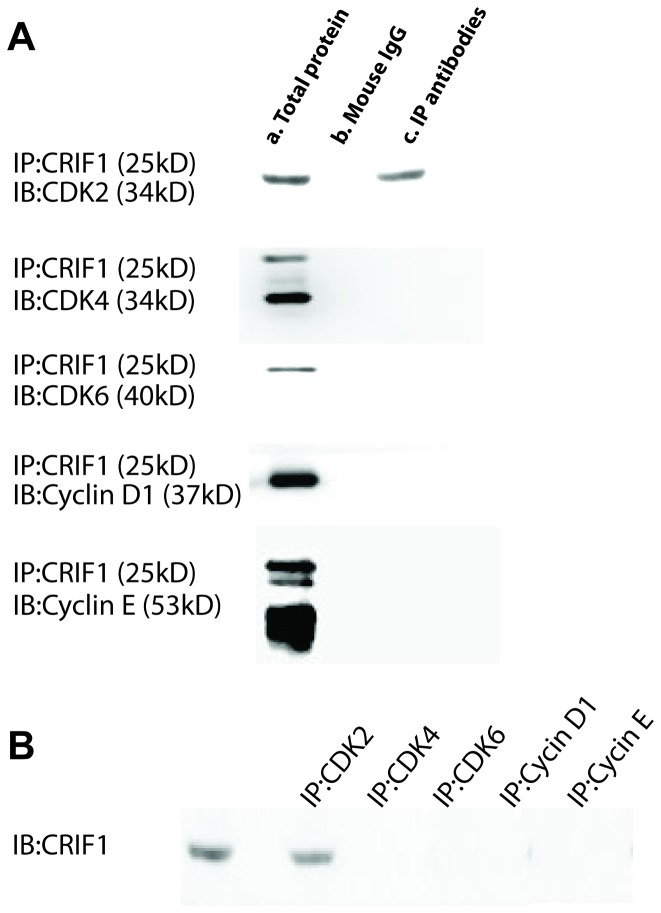
Interaction between CRIF1 and the key molecules in G1/S checkpoint. (**A**) Co-immunoprecipitation experiments (IP) were performed with CRIF1 agarose and complexes were analyzed in immunoblots (IB) with antibodies against CDK2, CDK4, CDK6, Cyclin D, and Cyclin E, respectively. a, Total protein from Jurkat cells (no antibody were added, positive control); b, agarose beads and normal mouse IgG (negative control); c, agarose beads and corresponding IP antibodies. (**B**) IP experiments were performed with antibodies against each protein and complexes were analyzed with CRIF1 antibody.

Cyclins and CDKs are two critical classes of molecules in determining the cell cycle progression. In order to investigate the key molecule(s) interacting with CRIF1 in the G1/S checkpoint, we used co-immunoprecipitation to detect the protein that binding to CRIF1 in Jurkat cells co-cultured with BMSCs. At 24 h after co-cultured, the proteins from Jurkat cells were extracted for co-immunoprecipitation using antibodies against CDK2, CDK4, CDK6, Cyclin D and Cyclin E, respectively. Immunoprecipitation result showed that CRIF1 only bound to CDK2 but not other examined proteins (**Figure 4A and B**).

### Expression level of CRIF1 in Jurkat cells affected G1 negative regulators

**Figure 5:**
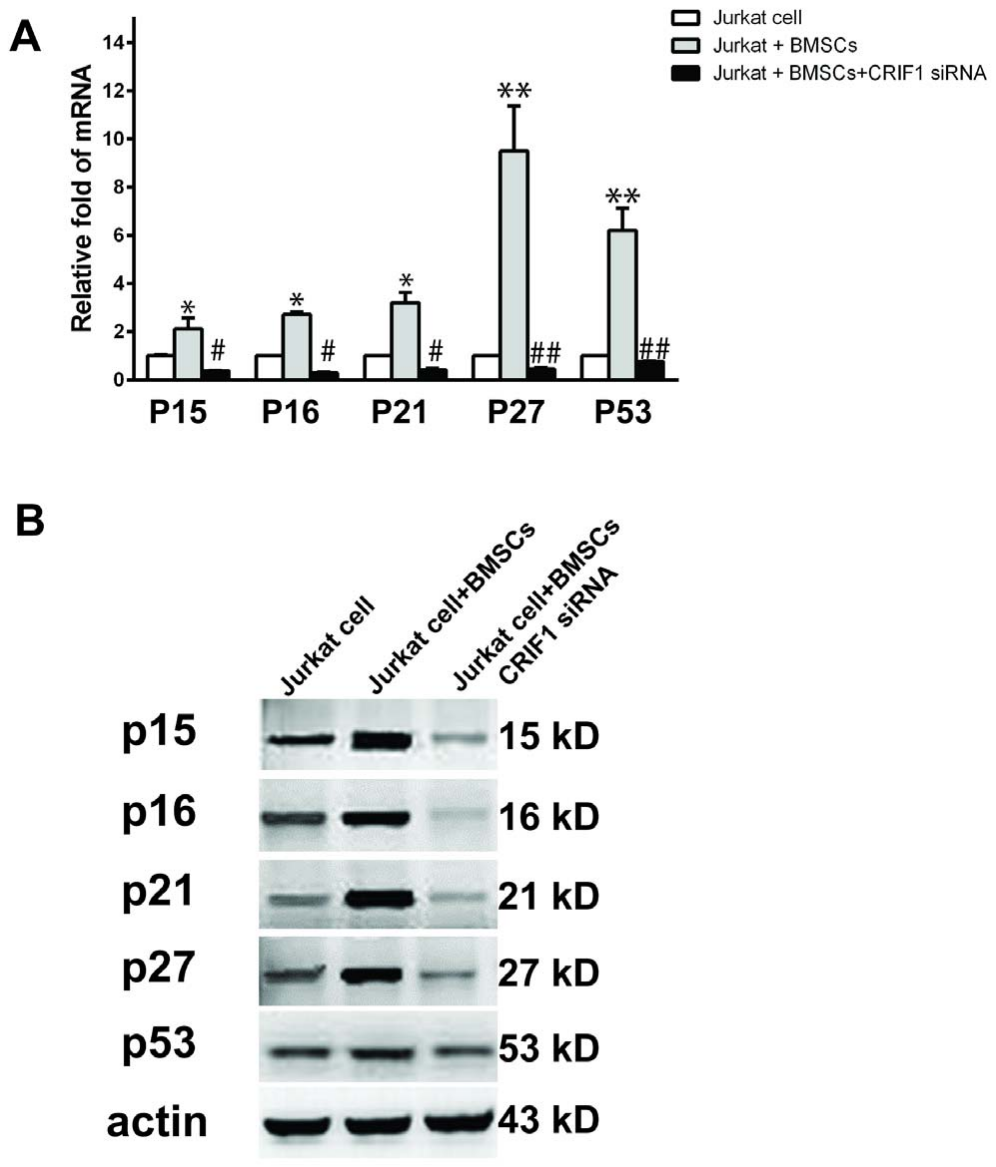
Detection of mRNA and protein levels of negative regulators. (**A**) mRNA level of P15, P16, P21, P27, and P53 in Jurkat cells, Jurkat cells with BMSCs co-culture and Jurkat cells with BMSCs co-culture and CRIF1 siRNA were measured with real-time PCR. (**B**) Detection of protein levels of above proteins in each condition. *p<0.05, v.s. Jurkat cells; ** p<0.01, v.s. Jurkat cells; #p<0.05, v.s. Jurkat cells with BMSCs; ##p<0.05, v.s. Jurkat cells with BMSCs.

CDKs play a key role in the process of G1 checkpoints. CRIF1 binding to CDK2 may arrest cell cycle through preventing the consequential signals of cell cycle. To further explore the possible involvement of the G1 negative regulators in the cell cycle arrest, we analyzed the levels of p15, p16, p21, p27 and p53 in Jurkat cells co-cultured with BMSCs. Quantitative real-time PCR result showed that BMSCs co-cultured significantly up-regulated mRNA levels of all negative regulators examined in this experiment (**Figure 5A**). WB result also showed similar effect of BMSCs co-culture on increasing protein levels of these regulators (**Figure 5B**). Interestingly, depletion of CRIF1 using siRNA inhibited BMSCs co-culture induced up-regulation of the negative regulators (**Figure 5A and B**). This result suggested that during BMSCs-induced cell cycle arrest increased CRIF1 might bind to CDK2 which resulted in the elevation of negative regulators and consequent arrest of cell cycle at the G1 phase. Depletion of CRIF1 might prevent the cell cycle arrest through inhibiting the expression of the negative regulators.

### Intracellular co-localization of CRIF1 and CDK2

**Figure 6:**
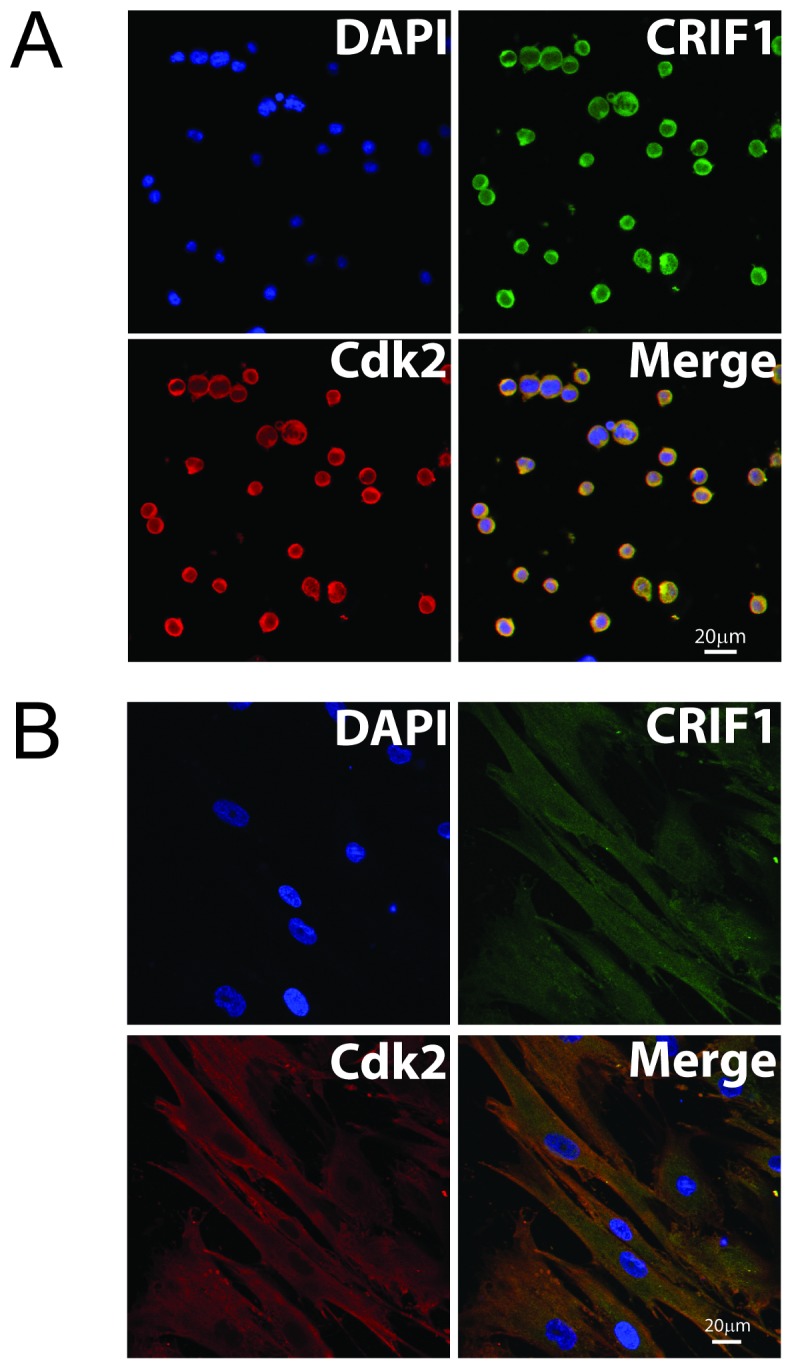
Cellular localization of CRIF1 and CDK2 in cells. (**A**) Jurkat cells co-cultured with BMSCs. (**B**) BMSCs. CRIF1 protein (green, FITC) and CDK2 (red, Cy3) were co-localized in both nuclear and plasma of Jurkat cells. Scale bar = 20 µm.

To explore the intracellular localization of CRIF1 and CDK2 in cells during cell cycle arrest, we performed double-immunofluorescence staining for CRIF1 and CDK2. Fluorescence signal for CRIF1 (green) and CDK2 (red) were detected using confocal microscopy in Jurkat cells co-cultured with BMSCs. The endogenous CRIF1 protein was detected in both nucleus and cytoplasm in Jurkat cells and it mainly located in the cytoplasm (**Figure 6A**). The distribution of CRIF1 in Jurkat cells observed in our experiment is consistent with previous report [24], Double-immunofluorescence staining revealed that the majority of CDK2 expression co-localized with CRIF1 in Jurkat cells (**Figure 6A**). Interestingly, similar intracellular co-localization and distribution of these two proteins were also observed in the BMSCs (**Figure 6B**).

## Discussion

CRIF1 was first discovered as a nuclear protein that acted as a negative regulatory factor in the cell cycle progression and cell growth [18]. Other biological functions of CRIF1 have also been reported, including its role in regulation of transcriptional activity through its DNA binding domain [25]–[29] and in promoting cell proliferation through interaction with CKII and the phosphorylation of CKBBP2/CRIF1 [30]. In this study, we reported for the first time that AML patients had a significantly decreased level of CRIF1 in their BM. Using an in vitro leukemia cell model, we found that overexpression of CRIF1 resulted in cell cycle arrest at G1 checkpoint in Jurkat cells. On the contrary, depletion of CRIF1 promoted cell cycle progress at the G0/G1 check-point and reversed BMSCs-induced cell cycle arrest in leukemia cells. This result suggested a regulatory role of CRIF1 in the progress of cell cycle in the leukemia cells. This role of CRIF1 might possibly exert through its interaction with CDK2 that consequently increased the levels of negative regulators, such as p15, p16, p21, p27 and p53, and ultimately lead to cell cycle arrest in the leukemia cells.

In this study, we included 40 BM samples from the healthy control and patients with AML or IDA. Decreased level of CRIF1 in the AML group and restored CRIF1 level in the AML-CR group suggested that the expression level of CRIF1 in the BM of patients might be associated with the disease status of AML. Compared to the healthy group, AML patients showed higher myeloproliferative activity that might be related to decreased level of CRIFI, a negative regulator of cell cycle.

Previously, we found that BMSCs co-culture can induce cell cycle arrest of Jurkat cells. This BMSCs-induced cell cycle arrest might be related to BMSCs-mediated negative regulatory mechanism. In this study, overexpression of CRIF1 resulted in the cell cycle arrest of Jurkat cells, while depletion of CRIF1 decreased cells arrested in G0/G1, indicating that inhibition of endogenous CRIF1 could promote cell cycle progression. Moreover, depletion of CRIF1 also reversed effect of BMSCs on the cell cycle arrest in leukemia cells, suggesting that CRIF1 might involve in the machinery of BMSCs co-culture induced negative regulation of G1/S check point of leukemia cells.

In the early G1 phase, cyclin D is expressed in response to a growth-promoting mitogen and subsequently binds to CDK4/6. The cyclin D/CDK4/6 complex is then activated by Cdk-activating kinase (CAK) which leads to the phosphorylation of the Rb protein. Phosphorylation of the Rb protein disrupts its association with the E2F family of transcription factors. The released E2F triggers the expression of several essential proteins for cell cycle progression, such as cyclin E and cyclin A. Cyclin E forms a complex with CDK2 and the activation of cyclin E/CDK2 complex completes the process by phosphorylating the Rb protein on additional sites. Cyclin/CDK complexes regulate progression through the G1 phase and the initiation of DNA synthesis or entry into the S phase [31], [32]. CDK is the center of cell cycle regulation and regulated by Cyclin and CKI. When combined with Cyclin, CDK is activated and promote cell proliferation, while binding with CKI prevents the cell cycle progression. Therefore, CDK is the key of G1/S regulation [33]–[35]. In this study, co-immunoprecipitation results showed that CRIF1 only interacted with CDK2, and suggested CRIF1 may act as CKI when combined with CDK2, and prevent cell cycle progression of leukemia cells. CRIF1 binding to CDK2 prevented the consequential signals of cell cycle, which caused G1 arrested and accumulations of G1 negative regulators, such as P15, P16, P21, P27, and P53 [36], [37]. Furthermore, BMSCs could up-regulate expression of certain genes by secretion of soluble cytokines. For instance, BMSCs induced expression of P27 in leukemia cells [11], and this effect was weakened by inhibition of endogenous CRIF1, and at the same time G1 negative regulators, such as P15, P16, P21, and P53 were all decreased. When the endogenous CRIF1 was inhibited, BMSCs could not induce cell cycle arrest and the cell cycle was allowed to progress through G1 phase, suggesting that CRIF1 might be a downstream factor of in response to the component derived from BMSCs.

Interestingly, previous studies reported that CRIF1 was exclusively localized in the nucleus in cells transfected with pMyc-CRIF1 or pEGFP-CRIF1 [18], [25], [26]. However, in our study we found that CRIF1 and CDK2 co-localized in both the nucleus and cytoplasm in Jurkat cells. Similar results were also observed in BM mesenchymal stem cells. We speculated that CRIF1 may partly translocate from the nucleus to the cytoplasm where it interacts with CDK2 and induces cell cycle arrest. CRIF1 might interact with CDK2 both in the nucleus and cytoplasm.

In summary, our results showed that CRIF1 overexpression arrested the cell cycle of leukemia cells, while depletion of endogenous CRIF1 reversed the BMSCs-induced cell cycle arrest. Specific interaction of CRIF1 with CDK2 was observed in both nucleus and cytoplasm in arrested Jurkat cells. These results suggest that decreased expression of CRIF1 in leukemia cells might contribute to the sensitivity of the leukemia cells to the chemotherapy drugs. However, it remains unclear how CRIF1 regulates the activity of CDK2 and where the binding cite is. Whether CRIF1 could be a key target to break dormancy of leukemia cells still need further investigation.
